# Acute Stroke in the Emergency Department: Profiles of Patients and Obstacles to Acute Intervention

**DOI:** 10.7759/cureus.64034

**Published:** 2024-07-07

**Authors:** Ashish N Bosco, Shakuntala Murthy, Girish Narayan, Karthik Reddy CH, Thomas Mathew, Raghunandan Nadig

**Affiliations:** 1 Emergency Medicine, St. John's Medical College, Bangalore, IND; 2 Neurology, St. John's Medical College, Bangalore, IND

**Keywords:** golden hour, window, thrombolysis, emergency department, stroke

## Abstract

Aims: To build a demographic profile of patients presenting to the emergency department (ED) with stroke, determine the proportion who successfully undergo thrombolysis and active interventions, and study their outcomes up to discharge or death in the hospital.

Methods and materials: A sample size of 215 was calculated and patients were recruited consecutively on presentation to the ED after obtaining consent. Data was collected and they were followed up till the outcome. Data was tabulated and analyzed both as a whole and after further categorization into infarction, hemorrhagic stroke, and cerebral venous thrombosis (CVT). Mean and standard deviation were used for continuous variables and chi-square for categorical variables.

Results: A total of 216 patients were recruited, 156 (72%) male and 60 (28%) female. There were 135 (63%) ischemic strokes, 67 (31%) hemorrhagic, and 14 (6%) CVT. The mean age was 56.57 years (SD 14.22 years). A total of 12 patients (5.5%) presented within the ‘golden hour’ and 28 ischemic strokes presented within the thrombolysis window, of which nine were thrombolyzed. In total, 39 patients were intubated in the ED, of which 10 (7.41%) had ischemic strokes, 27 (40.3%) had hemorrhagic strokes and two (14.29%) had CVTs. There were 192 patients admitted to in-patient care, while 24 (11%) were discharged against medical advice. A further 14 patients were intubated during admission. Nine patients (13.43%) with hemorrhagic strokes underwent surgical decompression, five (7.46%) had an external ventricular drain (EVD) placed, six (8.96%) underwent aneurysm clipping, and two (2.99%) underwent aneurysm coiling. One case of CVT underwent surgical decompression.

Conclusions: Stroke is a highly heterogeneous clinical entity with nuanced differences between the different subtypes. There appear to be significant obstacles regarding the early presentation of strokes to hospitals and the initiation of thrombolysis in the case of acute interventions.

## Introduction

Emergency medicine in India is still in early development; it was officially recognized as a specialization for postgraduate training in 2009. Urbanization increases the demand for skilled emergency personnel with expertise in managing a variety of acute clinical presentations [1]. The unique challenges of the Indian population, especially regarding a new specialty, emphasize the need for training in managing trauma and cardiovascular diseases [2].

South Asians (India, Pakistan, Bangladesh, Bhutan, Nepal, and Sri Lanka), constituting a quarter of the world's population, face significant health challenges such as premature atherosclerosis, high diabetes risk, and stroke prevalence. They experience disproportionately high stroke-related mortality, not fully explainable by socio-economic factors. In India, stroke incidence ranges from 123-145 per 100,000 annually in rural areas to 334-424 per 100,000 in urban areas. There is a critical lack of robust epidemiological data in South Asian countries, highlighting the urgent need for preventive measures, improved healthcare access, and collaborative research efforts to address these health disparities [3].

Community-based studies from India shed light on the unique profile of stroke in this population. India reports higher rates of hemorrhagic stroke (which is also associated with higher rates of mortality) compared to Western countries. Also, the prevalence of cerebral venous thrombosis (CVT) in South Asia is the highest in the world, accounting for a majority of strokes in young women in the region. Both stroke and coronary artery disease have been observed to occur on average, a decade earlier than seen in Western countries. In addition to this, studies in Western countries examining differences between different minority ethnic groups in stroke risk profile and prevalence, overwhelmingly show South Asians to be at much higher risk. This is especially true for young women, in whom strokes are common at a relatively young age [3].

Acute stroke, a subset of cardiovascular disease, is particularly significant in India, causing great distress among survivors and caregivers. The elderly fear the disabling effects of a stroke more than death itself [4].

Stroke has been conventionally defined as a neurological deficit of cerebrovascular origin persisting beyond 24 hours or resulting in death within 24 hours, covering subtypes like ischemic stroke, hemorrhagic stroke, and CVT [5]. However, this definition has faced scrutiny due to an improved understanding of the disease process. There is now a greater emphasis on the focal damage to the central nervous system (CNS) characteristic of a stroke. The previous 24-hour time limit has been reconsidered as significant and permanent damage can occur well within this arbitrary cut-off [5]. Advances in brain imaging, especially vascular imaging, widely available at most centers, contribute to this enhanced understanding.

As knowledge, technology, and personnel evolve, there is increasing clarity on the finer points of stroke pathophysiology and its management implications [6]. Recent studies on ischemic stroke highlight individual variations in the rate and reversibility of brain tissue damage resulting from infarction. The modern approach to acute stroke through brain imaging aims to differentiate already infarcted tissue from under-perfused but salvageable tissue, guiding therapy in the acute setting. This distinction is crucial not only for treatment decisions but also for differentiating a stroke from a transient ischemic attack (TIA), a transient neurological deficit without infarction [7].

In the emergency setting, when a patient displays symptoms of an acute stroke, clinicians must carry out time-sensitive actions that can significantly impact the disease course. A solid understanding of basic sciences and neuroanatomy is essential for diagnosis. Clinicians need to be aware of diverse presentations, including rare forms of the disease, and a thorough history and examination can provide insights into the stroke's anatomical site, possible underlying pathology, and mechanism [7].

Diagnosis of stroke heavily relies on imaging, with computerized tomography (CT) and magnetic resonance imaging (MRI) being the primary tools in acute cases. CT is more sensitive for hemorrhage, while MRI has higher sensitivity for infarction, particularly within the first 12 hours of presentation [7]. Angiography provides detailed information on specific vessel occlusion, sinus blockage, or the presence of an aneurysm causing a bleed, guiding further therapy and indicating the need for specialized interventions.

Modern emphasis leans towards multi-sequence MRI imaging for ischemic strokes, utilizing techniques like diffusion-weighted imaging (DWI), fluid-attenuated inversion recovery (FLAIR), and perfusion-weighted imaging to identify the 'penumbra'-tissue which could be hypo-perfused but salvageable. It is crucial to note that if the penumbra is not urgently re-perfused, it can also become infarcted [8].

The hemorrhagic subtype of stroke, while less common, holds significant public health importance due to its association with high morbidity and mortality [8-9]. Intracerebral hemorrhage exhibits a notably higher case fatality rate compared to other stroke subtypes [10]. In contrast to ischemic stroke, hemorrhagic stroke often presents with diffuse and non-specific symptoms. Similar to ischemic stroke, imaging, particularly CT and MRI, is indispensable for diagnosing intracerebral bleeding and is known for its high sensitivity in detecting bleeds, especially in acute cases.

It's crucial to clarify that traumatic intracranial bleeding falls outside the stroke definition. A subset of hemorrhagic strokes is caused by pre-existing vascular malformations, such as aneurysms. Detecting these malformations or lesions is vital, guiding further treatment. Patients with intracerebral hemorrhage (ICH) from these lesions are typically younger and tend to have better outcomes with prompt treatment. Recognizing the typical presentation and imaging pattern of bleeds in these cases is crucial for quick diagnosis in the emergency department (ED) [9].

CVT stands as the third subtype of stroke, distinguished by its unique characteristics. It encompasses thrombosis of the venous sinuses, draining cortical veins, or the deep venous system, leading to brain parenchymal damage through various mechanisms, including infarction due to venous stasis, hemorrhage due to back pressure, and focal edema [11]. Focal deficits may or may not result from CVT, with headache being the most consistent symptom, observed in approximately 80% of cases [12-13]. Other deficits manifest in correspondence to the area of potential infarction [14-15].

The clinical entity known as 'stroke' is notably diverse and intricate, comprising various subtypes with distinct characteristics. The ED serves as the primary point of contact for these patients. Here, the emergency physician makes a clinical diagnosis, conducts necessary imaging, and devises a treatment plan. The ED plays a pivotal role in time-sensitive decisions, enabling crucial interventions like thrombolysis or surgical decompression through the rapid response and clinical expertise of the team, in coordination with specialists. Data serves as the foundation for clinical skills, guiding patient evaluation and management.

Tertiary hospitals, especially those like ours with large catchment areas, receive a large number of patients with stroke. There is a high amount of 'atypical' cases as well, which don't fall into the classical archetype of stroke presentation. These cases can be missed on initial evaluation, leading to delays in diagnosis and initiation of treatment. Further, it has been observed that a higher proportion of 'younger patients' (below 50 years of age) were presenting with strokes. This questions conventional teaching about which age groups should be suspected to be at risk for strokes, meriting a more organized study of this occurrence. Patients often present to our hospital ED after traveling several hours over long distances, often crossing state lines. This impacts the feasibility of interventions like thrombolysis. Anecdotal observations over the years suggested that this was a significant problem meriting further study. 

The Indian setup faces numerous challenges in providing care to patients suffering from strokes. Patients incur significant out-of-pocket expenditures for healthcare, which is an often insurmountable barrier to receiving crucial (but expensive) treatments such as thrombolysis. Patients often travel long distances to seek care, losing the precious window when these interventions can be carried out. Awareness is yet to penetrate all sections of the Indian public regarding the signs of a stroke and the need to seek urgent help. With a limited number of centers offering comprehensive stroke care, getting a patient to a stroke center within the golden hour is a constant challenge, one that requires careful attention in the battle against this new epidemic of the developing world [1]. 

This study aims to contribute to the existing body of data on stroke incidence and presentation in India, offering a unique ED perspective on the diagnostic and therapeutic challenges associated with stroke. Focusing on our hospital, with a large catchment area spanning three neighbouring South Indian states, the study sought to profile stroke patients, identifying any discernible trends. Additionally, it aimed to analyze patient outcomes to discern trends that can inform ED decision-making. The goal was to enhance understanding and facilitate more informed care for stroke patients in the ED. 

The unique factors associated with stroke in the Indian population are the chief starting point for the study. By looking at the type, clinical presentation, and demographic characteristics of patients presenting to the ED with stroke, we aim to identify comparability to existing literature and contribute to the body of data on the presentation of stroke in India. Important unique findings would certainly merit further study. This would enable faster detection of stroke among atypical and obscure presentations, lowering the threshold for suspicion of stroke among younger patients, and refining decision-making about disposition which is predominantly based on clinical gestalt. Data on outcomes of patients with stroke who are admitted to in-patient care allows emergency physicians to better counsel and brief family members or other surrogate decision-makers about prognosis, and if necessary - to temper expectations of full functional recovery - a challenging task in the ED. Communication and health education are pillars of public health. Local data on stroke presentation and outcome could drive more targeted and specific public education on stroke and its management.

Another objective was to examine the apparent obstacles to the initiation of critical, time-sensitive interventions such as thrombolysis with rTPA (recombinant tissue plasminogen activator) and endovascular therapies. As stated earlier, these are known to significantly alter outcomes. However, public awareness of the need for urgency in seeking care in the event of a stroke is still limited. The high cost of these treatments may also be an obstacle for the Indian public.

## Materials and methods

The study was conducted over a period of two years (November 2019 - November 2021) following ethical clearance from the Institutional Ethics Committee. A sample size of 215 was calculated based on a study by Pandian et al. [2], with 5% absolute precision and 95% confidence level. The formula used is as follows:

$n=(Z^{2}_{1-\alpha/2} p(1-p))/d^{2}$

Where p = expected proportion, d = absolute precision, and $1-\alpha /2$ = desired confidence interval.

Cases were identified and recruited upon arrival at the ED using consecutive sampling, with consent obtained using a pre-approved consent sheet and an information sheet provided to the patient or their next of kin. Relevant data was collected from the emergency case sheet according to a pre-prepared proforma.

Data collected included demographic data such as age and sex. Other data collected included duration of hospitalization, symptoms on presentation, time since onset of symptoms in hours, deficits detected on clinical examination, patient comorbidities, intervention (if any) carried out in the ED, ED disposition, intubation in the ED or subsequently during in-patient care and final outcome after admission to hospital. No missing data was encountered.

**Figure 1:**
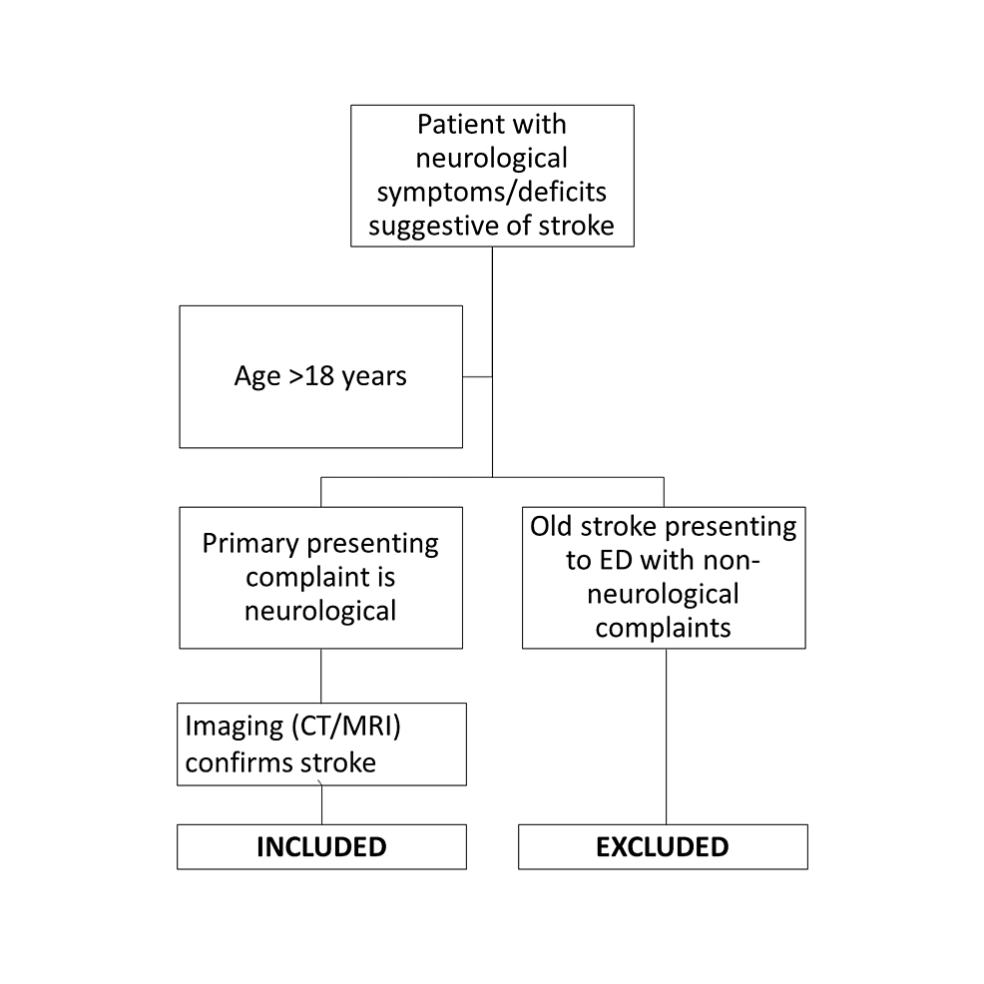
Flow chart of patient selection and exclusion criteria

Patients were then followed up until death or discharge from the hospital to collect outcome data and major in-hospital events from in-patient records and discharge summaries. Data was entered into a single spreadsheet in Microsoft Excel 2010 (Microsoft Corp., Redmond, WA), with additional spreadsheets created to separate the three subtypes of stroke (ischemic, hemorrhagic, and CVT) for further analysis. Inclusion criteria encompassed patients over 18 years presenting to the ED with primarily neurological complaints and diagnosed with stroke via radiological imaging (CT/MRI). Exclusion criteria included patients previously diagnosed with stroke (old stroke) presenting to the ED with non-neurological complaints. This is shown in Figure 1.

Statistical analysis was performed using Stata 17 (StataCorp LLC, College Station, TX), employing mean and standard deviation for continuous variables and chi-square for categorical variables. Correlation between stroke outcome and parameters such as age, co-morbidities, symptom nature and severity, imaging findings, and interventions was explored, with a p-value of <0.05 considered significant.

## Results

A total of 216 patients were recruited over a two-year period from 30 November 2019 to 30 November 2021. Consecutive sampling was employed. Of these, 156 (72.22%) were male and 60 (27.77%) were female. Out of the total 216, 135 (62.5%) suffered from ischemic stroke (referred to in tables and figures as ‘infarct’), 67 (31.09%) suffered from hemorrhagic stroke (referred to as ‘bleed’ in tables), and 14 (6.48%) were diagnosed with CVT. Table 1 breaks down the distribution of sex in each type of stroke. All three subtypes had a higher proportion of males, with 72.59% (n=98), 71.64% (n=48), and 71.43% (n=10) for ischemic strokes, hemorrhagic strokes, and CVT respectively. This finding, however, is not statistically significant.

**Table 1:** Distribution of sex with type of stroke The data has been represented as numbers (n), with percentages in brackets. The chi value is represented, with p<0.05 considered statistically significant. CVT: cerebral venous thrombosis

Sex	Infarct (n=135)	Bleed (n=67)	CVT (n=14)	Chi	P-value
Female	37 (27.41%)	19 (28.36%)	4 (28.57%)	0.025	0.988
Male	98 (72.59%)	48 (71.64%)	10 (71.43%)	0.025	0.988

The mean age of the data set was 56.57 years with a standard deviation of 14.22 years. There appears to be a statistically significant association between age and type of stroke. The association becomes clearer and more nuanced when the variable age is divided further into four categories, each spanning two decades. This is illustrated in Table 2. CVT showed the highest proportion of cases below the age of 40 (n=9, 64.29%), hemorrhagic stroke showed the highest proportion of cases aged 41-60 years (n=32, 47.76%), and ischemic strokes had the highest proportion of cases aged older than 60 years (n=66, 48.89%). These findings are statistically significant, with a p-value < 0.05 (Table 2).

**Table 2:** Distribution of age with type of stroke The data has been represented as numbers (n), with percentages in brackets. The chi value is represented, with p<0.05 considered statistically significant. CVT: cerebral venous thrombosis

Age (years)	Infarct (n=135)	Bleed (n=67)	CVT (n=14)	Chi	P-value
<20	0 (0.0%)	1 (1.49%)	2 (13.29%)	41.29	<0.050
21-40	12 (8.89%)	10 (14.93%)	7 (50.0%)	41.29	<0.050
41-60	57 (42.22%)	32 (47.76%)	3 (21.43%)	41.29	<0.050
>60	66 (48.89%)	24 (35.82%)	2 (14.29%)	41.29	<0.050

Table 3 shows the distribution of comorbidities with the type of stroke. There is an association between the type of stroke and the comorbidities of hypertension, diabetes mellitus, heart disease, and dyslipidemia. There is also a statistically significant association between post-partum state and the type of stroke.

**Table 3:** Distribution of comorbidities with type of stroke The data has been represented as numbers (n), with percentages in brackets. The chi value is represented, with p<0.05 considered statistically significant. CVT: cerebral venous thrombosis

Comorbidity	Infarct (n=135)	Bleed (n=67)	CVT (n=14)	Chi	P-value
Hypertension	98 (72.59%)	51 (76.12%)	3 (21.43%)	17.465	<0.05
Diabetes mellitus	71 (52.59%)	20 (29.85%)	3 (21.43%)	12.393	<0.05
Heart disease	26 (19.26%)	6 (8.96%)	6 (42.86%)	9.87	<0.05
Kidney disease	8 (5.93%)	8 (11.94%)	1 (7.14%)	2.447	0.326
Old stroke	17 (12.59%)	7 (10.45%)	0 (0.0%)	2.079	0.353
Connective tissue disorder	1 (0.74%)	1 (1.49%)	0 (0.0%)	0.415	0.812
Dyslipidemia	68 (50.37%)	13 (19.4%)	3 (21.43%)	19.988	<0.05
Hypothyroidism	7 (5.19%)	5 (7.46%)	2 (14.29%)	1.887	0.389
Polycythemia	2 (1.48%)	1 (1.49%)	1 (7.14%)	2.305	0.316
Peripheral vascular disease	1 (0.74%)	1 (1.49%)	0 (0.0%)	0.415	0.812
Malignancy	1 (0.74%)	1 (1.49%)	0 (0.0%)	0.4158	0.812
Anaemia	3 (2.22%)	0 (0.0%)	0 (0.0%)	1.925	0.401
Post-partum	0 (0.0%)	0 (0.0%)	2 (14.29%)	29.126	<0.05

Data on the initial clinical presentation in the ED was collected, with symptoms classified into 12 categories as shown in Table 4. There appears to be an association between the type of stroke and certain classical presenting complaints, with altered sensorium seen more often in bleeds with 25 cases (37.31%). Seizures (n=11, 78.57%) and headaches (n=11, 78.57%) were seen in a higher proportion of cases of CVT. Facial deviation (n=39, 28.89%), speech defects (n=60, 44.44%), and unilateral weakness (n=98, 72.59%) were present in the highest proportion among ischemic strokes. 

**Table 4:** Chief presenting complaints distributed by type of stroke The data has been represented as numbers (n), with percentages in brackets. The chi value is represented, with p<0.05 considered statistically significant. CVT: cerebral venous thrombosis

Presenting history	Infarct (n=135)	Bleed (n=67)	CVT (n=14)	Chi	P-value
Syncope	4 (2.96%)	4 (5.97%)	1 (7.14%)	1.346	0.510
Altered sensorium	22 (16.3%)	25 (37.31%)	0 (0.0%)	15.781	<0.05
Giddiness	21 (15.56%)	17 (25.37%)	2 (14.29%)	3.038	0.219
Seizure	7 (5.19%)	12 (17.91%)	11 (78.57%)	58.432	<0.05
Headache	7 (5.19%)	13 (19.4%)	11 (78.57%)	57.59	<0.05
Facial deviation	39 (28.89%)	9 (13.43%)	0 (0.0%)	10.4661	<0.05
Visual disturbance	8 (5.93%	1 (1.49%)	1 (7.14%)	2.207	0.332
Speech	60 (44.44%)	16 (23.88%)	1 (7.14%)	13.556	<0.05
Unilateral weakness	98 (72.59%)	29 (43.28%)	4 (28.57%)	22.57	<0.05
Nausea/vomiting	6 (4.44%)	18 (26.87%)	3 (21.43%)	21.671	<0.05
Sensory symptoms	2 (1.48%)	3 (4.48%)	1 (7.14%)	2.5446	0.280
Imbalance	7 (5.19%)	3 (4.48%)	0 (0.0%)	0.777	0.678

A total of 12 patients (5.56%) presented to the hospital emergency department within the so-called ‘golden hour’ for stroke management. Four of these cases were ischemic strokes while eight were hemorrhagic. Cases presenting to the ED within the golden hour have been categorized by type of stroke in Table 5. Hemorrhagic strokes had the highest proportion of cases presenting within the golden hour (n=8, 11.94%), with a p-value of <0.05 as seen in Table 5.

**Table 5:** Types of stroke presenting within the 'golden hour' The data has been represented as numbers (n), with percentages in brackets. The chi value is represented, with p<0.05 considered statistically significant. CVT: cerebral venous thrombosis

Type of stroke	Number presenting	Chi	P-value
Infarct (n=135)	4 (2.96%)	7.758	<0.05
Bleed (n=67)	8 (11.94%)	7.758	<0.05
CVT (n=14)	0 (0.0%)	7.758	<0.05
Total	12		

Ischemic strokes are associated with unique time ‘windows’ for important therapeutic interventions that bring about significant improvement in outcomes. The first is the window for thrombolysis with rTPA, taken as 4.5 hours. The second is the 24-hour mark, which is considered the window for mechanical thrombectomy; 28 (20.74%) cases of ischemic stroke presented to the ED within the thrombolysis window, and 53 (39.26%) cases presented to the ED within 24 hours of the onset of symptoms.

The initial presenting clinical findings (deficits) were also recorded on arrival. For the purpose of analysis, they have been classified into 17 categories as shown in Table 6. There appears to be a statistically significant association between the type of stroke and the findings of deficits in particular cranial nerves, with 90 (66.67%) of ischemic strokes showing deficits in cranial nerve three, while hemorrhagic strokes were more commonly associated with deficits in cranial nerve six (n=14, 20.9%), and cranial nerve seven (n=22, 32.84%). A similar association was found between the type of stroke and the finding of unilateral weakness (hemiparesis/hemiplegia), seen in 86 (63.7%) of cases of ischemic stroke. A higher proportion of 14 (20.9%) cases of hemorrhagic stroke presented with severe obtundation and could not have a detailed neurological examination performed, compared to 10 (7.41%) cases of ischemic stroke and one CVT (7.14%). This also appears to be statistically significant. 

**Table 6:** Neurological deficits by type of stroke The data has been represented as numbers (n), with percentages in brackets. The chi value is represented, with p<0.05 considered statistically significant. CVT: cerebral venous thrombosis

Deficit	Infarct (n=135)	Bleed (n=67)	CVT (n=14)	Chi	P-value
Consciousness	15 (11.11%)	14 (20.9%)	1 (7.14%)	4.153	0.125
Speech defect	35 (25.93%)	13 (19.4%)	1 (7.14%)	3.148	0.207
Cranial nerve 1	1 (0.74%)	0 (0.0%)	0 (0.0%)	0.602	0.740
Cranial nerve 2	0 (0.0%)	0 (0.0%)	0 (0.0%)	-	-
Cranial nerve 3	90 (66.67%)	31 (46.27%)	4 (28.57%)	12.912	<0.05
Cranial nerve 4	6 (4.44%)	2 (2.99%)	0 (0.0%)	0.843	0.656
Cranial nerve 5	12 (8.89%)	7 (10.45%)	0 (0.0%)	1.579	0.454
Cranial nerve 6	12 (8.89%)	14 (20.9%)	1 (7.14%)	6.294	<0.05
Cranial nerve 7	64 (47.41%)	22 (32.84%)	1 (7.14%)	10.785	<0.05
Cranial nerve 8	4 (2.96%)	2 (2.99%)	0 (0.0%)	0.4278	0.807
Cranial nerve 9	2 (1.48%)	0 (0.0%)	0 (0.0%)	1.2112	0.546
Cranial nerve 10	2 (1.48%)	0 (0.0%)	0 (0.0%)	1.2112	0.546
Cranial nerve 11	0 (0.0%)	0 (0.0%)	0 (0.0%)	-	-
Cranial nerve 12	0 (0.0%)	0 (0.0%)	0 (0.0%)	-	-
Hemiparesis/hemiplegia	86 (63.7%)	29 (43.28%)	4 (28.57%)	11.802	<0.05
Sensory deficit	4 (2.96%)	2 (2.99%)	0 (0.0%)	0.4278	0.807
Cerebellar signs	11 (8.15%)	7 (10.45%)	0 (0.0%)	1.67	0.434
Could not be assessed/severely obtunded	10 (7.41%)	14 (20.9%)	1 (7.14%)	8.2469	<0.05

A total of 39 patients were intubated in the ED. Of these, 10 (7.41%) were ischemic strokes, 27 (40.3%) were hemorrhagic and two (14.29%) were CVTs. Another 14 were intubated subsequently during the course of hospitalization, amounting to 53 (24.53%) patients in total. A higher proportion of hemorrhagic strokes (n=33, 49.25%) were intubated.

Admissions to our hospital involved three possible levels of care: the general ward for fully stable cases, the high dependency unit (HDU) for patients requiring continuous monitoring but not mechanical ventilation or high vasopressor support, and finally, the ICU for sick patients requiring mechanical ventilation or who are hemodynamically unstable. Clinician gestalt plays an important role in determining the level of care a patient requires at each stage of their stay in the hospital; 88 cases (40.74%) were admitted to the ICU, 49 cases (22.68%) to the HDU, and 55 (25.46%) to the general ward. Table 7 shows the breakdown of disposition by each type of stroke. Hemorrhagic strokes had the highest proportion of cases (n=49, 73.13%) admitted to intensive care, CVT had the highest proportion admitted to HDU (n=7, 50.0%) and ischemic strokes had the highest proportion (n=53, 39.26%) admitted to the general ward. A total of 24 (11.11%) of patients seen in the ED were discharged against medical advice (DAMA). Ischemic strokes had the highest proportion of cases that were DAMA (n=19, 14.07%). These findings are statistically significant.

**Table 7:** Patient disposition by type of stroke The data has been represented as numbers (n), with percentages in brackets. The chi value is represented, with p<0.05 considered statistically significant. CVT: cerebral venous thrombosis; HDU: high dependency unit; DAMA: discharged against medical advice

Disposition	Infarct (n=135)	Bleed (n=67%)	CVT (n=14)	Chi	P-value
ICU	34 (25.19%)	49 (73.13%)	5 (35.71%)	60.285	<0.05
HDU	29 (21.48%)	13 (19.4%)	7 (50%)	60.285	<0.05
Ward	53 (39.26%)	1 (1.49%)	1 (7.14%)	60.285	<0.05
DAMA	19 (14.07%)	4 (5.97%)	1 (7.14%)	60.285	<0.05
Total	135	67	14		

The mean duration of hospital stay was 17.45 days with a standard deviation of 50.33 days. Duration of stay was divided into three categories: up to two days, three to seven days, and beyond seven days, and studied for each type of stroke (Table 8). Ischemic strokes tend to fall in the second category (ie: 3-7 days), while hemorrhagic strokes tend to fall in the third, indicating a tendency for longer hospitalization. The association was found to be statistically significant as seen in Table 8.

**Table 8:** Hospital stay duration (in days) by type of stroke The data has been represented as numbers (n), with percentages in brackets. The chi value is represented, with p<0.05 considered statistically significant.

Duration (days)	Infarct (n=135)	Bleed (n=67)	CVT (n=14)	Chi	P-value
0-2	39 (28.89%)	12 (17.91%	3 (21.43%)	10.803	<0.05
3-7	53 (39.26%)	18 (26.87%)	4 (28.57%)	10.803	<0.05
>7	43 (31.85%)	37 (55.22%)	7 (50.0%)	10.803	<0.05

There was no statistically significant association between the type of stroke and final outcome (discharge, DAMA, or death in the hospital). Of the total 216 patients, 192 (88.88%) were admitted to the hospital. Of the 192 admitted patients, 139 (72.39%) were discharged, 38 (19.79%) were discharged against medical advice and 15 (7.81%) died in hospital. There was no statistically significant association between the type of stroke and final outcome (discharge, DAMA, or death in the hospital).

Of the total number of ischemic strokes represented, six patients (4.44%) were thrombolyzed with alteplase, and three (2.22%) were thrombolyzed with tenecteplase. The recommended window for the use of tenecteplase is three hours, while the window for alteplase is 4.5 hours. Combined decision-making from the ED team and the on-call neurologist guided the choice of drug. Only one patient (0.07%) underwent a mechanical thrombectomy, and two (1.31%) underwent surgical decompression. Six patients (4.44%) with ischemic stroke would go on to experience hemorrhagic transformation of the infarct confirmed by imaging. Nine patients (13.43%) with hemorrhagic strokes underwent surgical decompression, five (7.46%) had an external ventricular drain (EVD) placed, six (8.96%) underwent aneurysm clipping, and two (2.99%) underwent aneurysm coiling.

## Discussion

Of the total number of 216 patients, a higher proportion were men, accounting for 72% of the total. This seems to be in keeping with well-known trends observed in previous literature, including large global epidemiological data sets [16-17]. In the Indian setting, it is worth studying if social factors may also be at play concerning disparities in the accessibility of healthcare between the sexes. This data is however difficult to capture, especially in the acute setting.

As with global trends, ischemic strokes appear to be the most common type of stroke in patients presenting to the hospital. It is globally estimated that hemorrhagic strokes account for 15% of the total stroke burden. In our data set, the proportion of hemorrhagic strokes was nearly double that number at 31%. This appears to corroborate what other community-based studies in the country have found to be unique in the South Asian population, which estimates a 19-46% proportion of hemorrhagic strokes [8]. It is already well-known that the hemorrhagic stroke subtype lags behind ischemic stroke in terms of quality of evidence and consensus on best practices and critical decision-making [12]. A higher burden of hemorrhagic strokes makes it all the more crucial for more high-quality studies to improve lacunae in knowledge. Cases of CVT made up 6% of the data set, again reflecting existing data about the relatively higher prevalence of this subtype in the South Asian population [3].

It has been said that stroke types such as CVT are more common in women, especially in the younger age group [3]. However, this study found no statistically significant association between sex and the type of stroke with which the patient presented.

With a mean age of 56.57 years, our study population appears to be a relatively younger one. It is known that strokes tend to present a whole decade earlier in the South Asian population compared to Western countries. The problem of stroke in younger adults appears to be a significant one, reflected in our findings [14]. On further investigation, it appears that older patients tend to suffer from ischemic and hemorrhagic strokes while CVT tends to be a disease of younger patients. Ischemic strokes were found to occur more in the seventh decade of life, while hemorrhagic strokes were found to occur more in middle age in the fifth and sixth decades of life. By contrast, over half of the cases of CVT were below the age of 40 - reinforcing the above point. The takeaway is that one cannot dismiss the diagnosis of stroke in a younger patient when there is ambiguity in clinical assessment.

Stroke is one of the most heterogeneous clinical entities, and this is perfectly exemplified by the wide array of clinical signs and symptoms that a stroke patient may present with. It is important to keep a low threshold for clinical suspicion of a stroke so as not to miss a potential diagnosis. The truly outcome-modifying treatment modalities available are time-sensitive, making early diagnosis absolutely crucial.

Beyond identifying a potential stroke, the clinical signs and symptoms can also help to refine the diagnosis by pointing towards a particular subtype. This allows a more focused evaluation, with more efficient use of resources. Classical teaching tells us that hemorrhagic strokes tend to present with sudden onset and rapid progression of symptoms [5]. Further, we found that hemorrhagic strokes tended to present more frequently with altered sensorium and depressed consciousness. Patients with hemorrhagic stroke tend to present in comatose or severely obtunded states more often in proportion to the other subtypes. Nausea and vomiting were also more frequently seen with this subtype. The well-known stroke symptoms of unilateral weakness and facial asymmetry tended to be seen more commonly with ischemic strokes.

A large majority of cases of CVT presented with headache, sometimes as the only complaint. This appears to be typical of CVT across multiple studies examining the same [12]. Headache is one of the most difficult complaints to evaluate in the ED. A diagnosis of CVT should always be kept in mind by the emergency physician in the Indian setting, especially in younger patients with suspicious patterns of headaches such as a ‘thunderclap headache’ or a headache described by a patient as the ‘worst’ of their life. Similarly, a presenting complaint of seizures, in the context of stroke, seems to be significantly associated with CVT more than the other subtypes. A low threshold for ordering magnetic resonance or CT venography is therefore worth considering in patients with this presentation. This will of course require balancing with the principles of reasonable resource use that govern healthcare. More study is recommended on the subject. The vast majority of other neurological complaints appear to have no predilection for any particular type of stroke. Stroke should be a prominent differential in patients presenting with a wide variety of neurological complaints.

Clinical examination remains crucial in evaluating a stroke, even in the emergency setting, frequently helping to confirm or further qualify the diagnosis. Examination findings will sometimes provide clues to the etiology and subtype of stroke. It was found that the third cranial nerve was involved most commonly with ischemic strokes, as was the seventh cranial nerve. Features of sixth cranial nerve palsy were found proportionally higher with hemorrhagic strokes. Hemorrhagic strokes also tended to present more frequently with depressed consciousness or somnolence, similar to findings of other large descriptive studies of strokes [11]. The classical stroke finding of hemiparesis/hemiplegia was most commonly associated with ischemic strokes. Findings like these are quite intuitive and seem obvious thanks to accepted knowledge. A few of our more obscure findings, however, are important to note for future reference when encountering such cases.

Several comorbidities have been proven to increase the risk of stroke beyond doubt. Hypertension is the most prominent, and its link to stroke is reaffirmed extensively in the literature [5]. In total, 152 patients (70.37%) out of 216 were known to be hypertensive or diagnosed with hypertension upon admission. Hypertension also appears to be a risk factor in a higher proportion of ischemic and hemorrhagic strokes. When comparing younger and older patient groups, hypertension appears to contribute to a higher proportion of strokes in patients over the age of 40.

Diabetes was also a risk factor in a proportionally higher number of ischemic strokes, as was dyslipidemia. Heart disease appeared to have a significant association with CVT. There doesn’t appear to be any precedent for this in literature, except for reports of this association seen in neonates [14]. This evidence, however, refers to congenital heart disease and is unlikely to be comparable to the disease process in adult patients, and larger studies will be required to frame definite conclusions on the matter.

Perhaps the most important metric to study strokes from a therapeutic standpoint is the time to presentation in the ED from the onset of symptoms. The ‘golden hour’ finds frequent mention in literature, and patients of stroke presenting within this first hour after onset of symptoms, have been found to have consistently better outcomes than those who present after this period of time [18]. In our study group of 216 cases, only 12 (5.56%) presented to the ED within one hour - a deeply concerning statistic. A significantly higher proportion of hemorrhagic strokes appeared to present within this time cut-off. This is possibly due to the sudden onset and rapid progression of symptoms in a hemorrhagic stroke and the higher frequency of depressed consciousness on presentation in this subtype. Such symptoms would be deeply alarming to family members or bystanders, prompting a quicker response and transport to the hospital.

The time since onset is of particular importance in the presentation of an ischemic stroke since outcome-changing interventions like thrombolysis and endovascular procedures are time-dependent, providing benefit only when carried out within strict time cutoffs. Attempting them beyond these cutoffs has been clearly demonstrated to be not just counter-productive, but downright dangerous [19]. Out of 135 ischemic strokes, only 28 (20.74%) presented to the ED within the window for thrombolysis with rTPA. This is a troubling finding since our center is one of very few in the city or even state which boasts a fully-fledged ‘stroke unit’, involving multiple specialties and offering the full spectrum of acute care in stroke. Even more worrying is the finding that only 39.26% of our patients with ischemic strokes presented to the ED within 24 hours of onset, which is considered the upper limit permitted for a mechanical thrombectomy [7]. The fact that barely a fifth of all ischemic strokes presented in time for thrombolysis and barely a third in time for mechanical thrombectomy is unfortunate. This represents a serious obstacle to proper stroke care and merits separate investigation. Our hospital provides health services to patients from a wide catchment area that spans three neighboring states. It is possible that the long travel times required to reach a tertiary hospital may be partly responsible for the delay in presentation. A lack of awareness in the community might also contribute. In the absence of proper basic health education, the need to seek urgent care in the event of stroke-like symptoms may be lost on the general population. The lack of awareness about newer treatment modalities might also be significant, with damage control rather than reperfusion being the cornerstone of stroke management for decades before the relatively recent emergence of thrombolysis and endovascular therapies. Public awareness may not yet have grasped the time-sensitive nature of these interventions, and the potential for significant improvement in outcomes when they are appropriately carried out on patients. This has been found to be a problem in other studies on the topic [19].

Further, only nine of the possible 28 cases were thrombolysed with rTPA. This amounts to 6.66% of all ischemic strokes. This is below average in comparison to the numbers observed Indo-USA National Collaborative Stroke Registry [18]. While there are clearly defined contraindications and certain circumstances where it is prudent not to go ahead with rTPA administration, the prohibitively high cost of the drug certainly puts it out of reach of a large proportion of the population. Private hospitals do not have the means to subsidize the cost of thrombolytic agents. Meanwhile, public health systems in the country are even more poorly equipped for acute stroke care, to the detriment of the large part of the population that is dependent on them for healthcare [19]. Only 1 out of a possible 53 cases presenting within 24 hours underwent a mechanical thrombectomy. Here again, it is certainly possible that the procedure might not have been indicated in several cases based on the pattern of infarct and vessel occlusion. But the fact remains that in a health system where much of the cost is borne out of the patient’s pocket, a procedure like a mechanical thrombectomy is prohibitively expensive. Financial constraints will certainly play a massive role in the rates of administration of these expensive drugs and interventions. It has been established that India falls behind significantly with the implementation of these acute stroke interventions [20].

Close to a quarter (24.53%) of our stroke patients were intubated at some point in the hospital. Of those, 39 (18.05% of the total) required emergency intubation on arrival. Hemorrhagic strokes tended to get intubated proportionally more than the other subtypes. This may be explained by the sudden and rapid development of symptoms in a hemorrhagic stroke, coupled with the higher proportion of hemorrhagic strokes that present with depressed consciousness and potentially require more emergent airway protective measures.

Following the initial evaluation and treatment carried out in the ED, the decision for disposition is made in conjunction with the neurology and neurosurgical teams, as applicable. Patients may be admitted to the ICU if intubated or otherwise unstable and critically ill. Cases requiring operative management or interventions like mechanical thrombectomies and thrombolysis also get admitted to the ICU thereafter. Patients who are relatively stable but in need of more regular monitoring are admitted to the HDU. Finally, those who are stable and managed medically are admitted to the ward. A significantly higher proportion of hemorrhagic strokes were admitted to the ICU from the ED, possibly a reflection of the relatively severe deficits at presentation. Conversely, a higher proportion of ischemic strokes were admitted to the wards of the hospital. A possible reason for this is the relatively gradual progression of the pathology. 50% of patients with CVT were admitted to the HDU. This implies that while not overtly unstable, these patients did require a degree of monitoring - likely a result of the varied and unpredictable presentation that CVT is known for. Around 11.11% of patients overall were discharged from the ED against medical advice. The reasons for DAMA are many - including financial constraints, a refusal of admission, a preference for treatment at another hospital, or a preference to seek treatment in the patient’s native place. A higher proportion of patients with ischemic stroke sought discharge against medical advice. Here again, public perceptions come into play. It has already been mentioned that over half of our patients with strokes presented beyond 24 hours after the onset of symptoms. While not candidates for active intervention, they certainly require admission for evaluation, initiation of medications to prevent worsening and recurrence, and continued observation. Perhaps a combination of lacking health education about the crippling long-term effects of a stroke, and the apparent indolent course of ischemic strokes result in patients and their families not prioritizing admission. Public health awareness is key in this regard because the initiation of even simple measures like anti-platelets and statins improves long-term outcomes and prevents recurrence. Rehabilitative measures initiated in-hospital also improve functional outcomes in patients rendered disabled by a stroke [5].

Hemorrhagic strokes tended to have a longer duration of hospital stay, with over half (55.22%) admitted for seven days or more in the hospital. This is in keeping with the recurring theme of hemorrhagic strokes tending to present with more severe symptoms and more frequently requiring critical care. A high proportion of cases of CVT (50.0%) also had hospital stays of seven days or more. Patients with ischemic stroke tended to have a shorter duration of hospital stay, a majority staying in hospital for less than seven days. Ultimately, the majority of patients in the study group ended up being discharged in a stable condition for follow-up (n=139, 72.39%). A small proportion, with no predilection for the type of stroke, were discharged against medical advice (n=38, 19.79%). A total of 15 patients (6.94%) died in the hospital.

We acknowledge certain limitations of our data set and study. The numbers, especially for hemorrhagic and CVT, aren’t high enough to make conclusions applicable to the population as a whole. At best, some of our findings may be considered hypothesis-generating and meriting further study. A small part of our data was collected during the initial months of lockdown in the earliest days of the COVID-19 pandemic, and the impact of those extraordinary circumstances on our data set is difficult to quantify. As with any observational study, there is tremendous inter-observer variability in assessing numerous parameters such as clinical assessments and decisions regarding disposition. The stroke team at our hospital is firmly rooted in guidelines and best evidence. However, variability may still exist in the finer points of acute stroke management that may impact data. The number of patients who are discharged against medical advice from both the ED and also in-patient care represents a significant loss of data that certainly impacts findings, but is an inevitable reality that has to be accounted for in any study [21].

## Conclusions

Stroke represents a common yet highly diverse presentation in the ED, necessitating a comprehensive understanding of associated signs, symptoms, and different stroke variants. Recent advancements in acute ischemic stroke management emphasize acute reperfusion strategies beyond mere damage control, underscoring the critical importance of familiarity with treatment modalities for emergency physicians. However, significant obstacles hinder the initiation of key interventions, warranting further investigation. Delays in stroke presentations to the ED further exacerbate reperfusion limitations, highlighting the urgent need for improved public awareness regarding the urgency of seeking care during a stroke. Given the significant public health burden posed by strokes, particularly in tertiary hospitals, there is a pressing need to enhance stroke services, including the development of multi-specialty stroke teams to optimize outcomes. Cerebral venous thrombosis, a less understood stroke variant, presents unique challenges due to its unpredictable presentation and clinical course, necessitating further research to enhance understanding and evidence quality. Despite strokes' longstanding presence in medicine, ongoing learning and research remain crucial for advancing treatment efficacy and fostering innovation in stroke care.
